# CRISPR/Cas9-mediated gene disruption determines the roles of MITF and CITED2 in human mast cell differentiation^∗^

**DOI:** 10.1182/bloodadvances.2023012279

**Published:** 2024-06-06

**Authors:** Jiezhen Mo, Fredrik Wermeling, Gunnar Nilsson, Joakim S. Dahlin

**Affiliations:** 1 Department of Medicine Solna, Center for Molecular Medicine, Karolinska Institutet and Karolinska University Hospital, Stockholm, Sweden; 2 Department of Medical Sciences, Uppsala University, Uppsala, Sweden

**TO THE EDITOR:**

The differentiation of hematopoietic stem and progenitor cells into the various cell lineages is regulated by the progenitors’ microenvironment and cell-intrinsic factors,^1^, ^2^, ^3^ including transcription factors and transcriptional modulators. The cell-intrinsic factors that regulate mast cell differentiation have primarily been studied in mouse hematopoiesis.^4^, ^5^, ^6^ An elegant example is the antagonistic regulation of MITF and C/EBPα, transcription factors that determine whether bipotent basophil/mast cell progenitors form mast cells or basophils.^7^ Technological advancements in genetic editing, including the CRISPR/Cas9 system, offer an attractive solution to knockout genes of interest in human hematopoietic progenitors, thus allowing for studies of the genes’ role in human hematopoiesis. Here, we aimed to develop a framework that allows for investigation into the role of individual transcription factors and transcriptional modulators in human mast cell differentiation.

We first optimized the CRISPR/Cas9-based method by genetically disrupting the *PTPRC* gene in hematopoietic progenitors from peripheral blood (supplemental Methods). The *PTPRC* gene encodes the cell surface protein CD45, which allows for a simple flow cytometry–based readout of the editing efficiency after culture. Ribonucleoprotein (RNP) of Cas9 and single guide RNA (sgRNA) targeting the *PTPRC* gene^8^ were prepared and delivered to 2-day cultured hematopoietic progenitors through electroporation (Figure 1A). Titration with increasing amounts of Cas9 RNP showed that the editing efficiency reaches a plateau at 840 pmol, achieving a mean editing efficiency of 74.8% with no or little effect on cell survival (Figure 1B-C; supplemental Figure 1). Therefore, the 840 pmol or 960 pmol amount of Cas9 RNP was used in the following experiments unless otherwise specified in the figure legends. To assess the editing efficiency in mast cells derived from CD34^+^ progenitors, we cultured the RNP-treated cells for 11 to 12 days after electroporation. Flow cytometry analysis revealed a mean editing efficiency of 66% in the c-Kit^hi^ FcεRI^+^ mast cells across 5 independent experiments (Figure 1D-E). Thus, the results demonstrate that CRISPR/Cas9–based gene editing can efficiently knock out a gene of interest in hematopoietic progenitors that are differentiating to cells of the mast cell lineage.

**Figure 1.**
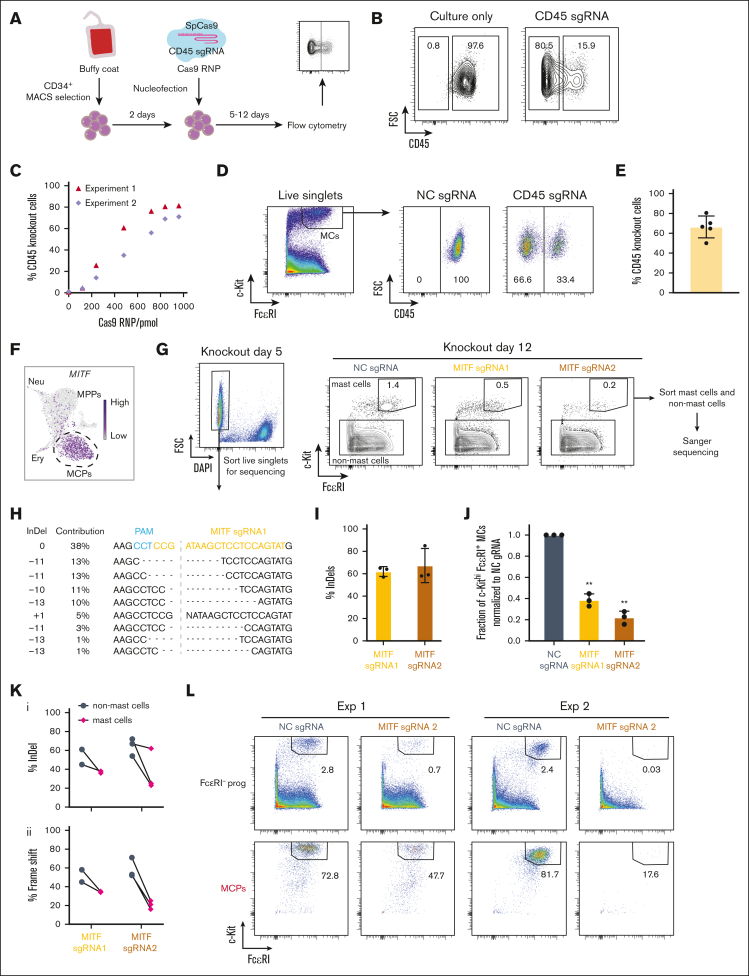
**MITF is required for human mast cell differentiation.** (A) Overview of the CRISPR/Cas9 RNP knockout strategy. (B) Representative flow cytometry plots showing the ablation of CD45 5 days after electroporation. Cells gated on live singlets. (C) The percentage of CD45-deficient cells with increasing doses of Cas9 RNP. Two independent experiments are shown. Analysis of cells by flow cytometry 5 days after electroporation. (D) Representative flow cytometry plots showing the frequency of CD45^–^ and CD45^+^ mast cells after genetic knockout. (E) The frequency of CD45-deficient mast cells assessed by flow cytometry. Analysis was conducted on day 11 or day 12 after electroporation, with Cas9 RNP dosages of 780 to 960 pmol. Each dot in panel E represents 1 independent experiment. (F) Single-cell RNA-sequencing data visualized using the UMAP embedding. The gene expression level of *MITF* is plotted in the hematopoietic progenitor landscape. MPPs, neutrophil (Neu) progenitors, erythoid (Ery) progenitors, and MCPs are annotated. (G) The left panel shows a representative flow cytometry analysis plot of in vitro–differentiated CRISPR/Cas9-modified CD34^+^ progenitors 5 days after electroporation with MITF sgRNA. The right panels show representative flow cytometry analysis plots of the cells 12 days after electroporation. Cells gated on live singlets. (H) Example sequencing results of the sorted live cells after electroporation with MITF sgRNA1. Data generated by the Inference of CRISPR Edits (ICE) software based on the Sanger sequencing results of the polymerase chain reaction amplicon. (I) Gene editing efficiency shown as percentage of insertions-deletions (InDels) of MITF sgRNA1 and sgRNA2 in live cells sorted 5 days after electroporation. (J) Fraction of c-Kit^hi^ FcεRI^+^ mast cells normalized to NC (negative control) sgRNA in live singlets 12 days after electroporation. Two-tailed 1 sample *t* test, hypothetical value is 1. The NC sgRNA condition refers to nontargeting sgRNA. The cells were cultured without interleukin-3 (IL-3) during the second week, apart from in 1 of the independent experiments in which IL-3 was present. (K) Editing efficiency calculated by percentage of InDel (Ki) and percentage of frameshift (Kii) in the mast cell population and the non-mast cell population within individual samples. Cells were analyzed 12 days after electroporation. The editing efficiency was analyzed in 2 independent experiments using MITF sgRNA1 and 3 independent experiments using MITF sgRNA2. (L) Flow cytometry plots showing the 2 outputs of sorted FcεRI^–^ progenitors and MCPs 12 days after electroporation with MITF sgRNA2, followed by differentiation into mast cells. Cells gated on live singlets. DAPI, 4′,6-diamidino-2-phenylindole; FSC, forward scatter; MACS, magnetic-activated cell sorting; MC, mast cell; NC, negative control; PAM, protospacer adjacent motif.

To identify candidate regulators of human mast cell differentiation, we analyzed a single-cell transcriptomics data set of human hematopoietic progenitors.^9^ Uniform Manifold Approximation and Projection (UMAP) visualization of the data revealed that the mast cell progenitor (MCP) population expressed the transcription factor *MITF* to a higher extent than multipotent progenitors (MPPs, Figure 1F). Therefore, we hypothesized that MITF drives the formation of mast cells in human hematopoiesis. To knock out *MITF* in CD34^+^ hematopoietic progenitors, 2 unique sgRNAs were designed to target near the 5’end of *MITF* (supplemental Figure 2A). We validated the editing efficiency of *MITF* 5 days after electroporation using an established Sanger sequencing–based approach, which has been developed to genotype cells in situations in which a flow cytometry–based readout to assess the presence or absence of a target is difficult or impossible.^10^ Analysis of sorted live cells showed an overall gene knockout efficiency of 62% for MITF sgRNA1 and 67% for MITF sgRNA2, indicating that *MITF* was successfully targeted (Figure 1G-I). Flow cytometry analysis 12 days after the gene editing event revealed a lower frequency of mast cells in *MITF*-targeted cells than control cells (Figure 1G,J). These data supported the hypothesis that MITF is important for human mast cell formation. Because a low frequency of mast cells was present 12 days after electroporation (Figure 1G), we investigated the genotype of the non-mast cell and the mast cell populations specifically. As expected, sorted non-mast cells showed a high degree of frameshift mutations in *MITF*, which disrupt the translation of the *MITF* RNA (Figure 1K). By contrast, we observed a low fraction of sequences with frameshift mutations in the mast cell population (Figure 1Kii). The low mast cell frequency and low fraction of mast cells with *MITF* frameshift mutations highlight that hematopoietic progenitors that have escaped the gene editing event are the main contributors to the mast cell population, stressing the importance of MITF for human mast cell formation.

We performed 2 additional experiments to investigate the effect of knocking out MITF in FcεRI^–^ progenitors and MCPs (supplemental Figure 2B).^9^ Analysis of the editing efficiency showed successful gene editing 5 days after electroporation from the 2 starting populations (supplemental Figure 2C). Knocking out MITF in sorted FcεRI^–^ progenitors and MCPs resulted in a lower frequency of mast cells on day 12 than the corresponding control wild-type progenitors in both experiments (Figure 1L). The low number of mast cells after culture in the knockout conditions made it difficult to assess the genotype of the remaining cells 12 days after gene editing (Figure 1L). Still, we were able to quantify the editing efficiency in the mast cell population in one of the experiments (supplemental Figure 2D). Notably, the sequencing data from this experiment showed that the mast cells at day 12 had a low degree of frameshift mutations, especially among mast cells derived from the sorted MCP population (supplemental Figure 2D). These results reiterate that mainly cells that have escaped the gene editing event form mast cells. Taken together, the results show that MITF is important for the human mast cell differentiation.

Single-cell RNA-sequencing analysis of hematopoietic progenitors revealed that the gene expression of the transcriptional modulator *CITED2* is gradually increasing along the MPP to MCP differentiation trajectory (Figure 2A).^9^ This observation prompted us to investigate the role of CITED2 for the formation of mast cells. Three sgRNA targeting near the 5’ end of *CITED2* were designed (supplemental Figure 3). CD34^+^ hematopoietic progenitors were subjected to CRISPR/Cas9-based gene editing, and sorted live cells were analyzed on day 5 for editing efficiency. CITED2 sgRNA1 showed a high degree of editing (supplemental Figure 3C) and was therefore used in subsequent experiments. Targeting *CITED2* in the hematopoietic progenitor population did not reduce the mast cell frequency (Figure 2B-D; supplemental Figure 3B-D). Instead, there was a trend that the knockout of *CITED2* results in an increased mast cell frequency (Figure 2D; supplemental Figure 3D), highlighting that mast cells are formed in the absence of CITED2. The Sanger sequencing analysis revealed successful knockout of *CITED2* in the mast cell population (90.5 % [mean] frameshift mutations), further highlighting that mast cell differentiation is not dependent on *CITED2* (Figure 2E). Additional experiments in which *CITED2* was genetically disrupted in FcεRI^–^ progenitors and MCPs confirmed the dispensable role of *CITED2* for the formation of mast cells (Figure 2F-H). Follow-up studies using the mast cell lines LAD2 and HMC-1 demonstrated that wild-type and *CITED2*-deficient mast cells survived equally well and proliferated with similar kinetics (Figure 2I-J). Taken together, our results collectively suggest that CITED2 is dispensable for mast cell differentiation, survival, and proliferation. Whether the knockout of *CITED2* alters the phenotype or function of the mast cells or the terminal mast cell maturation has yet to be investigated.

**Figure 2.**
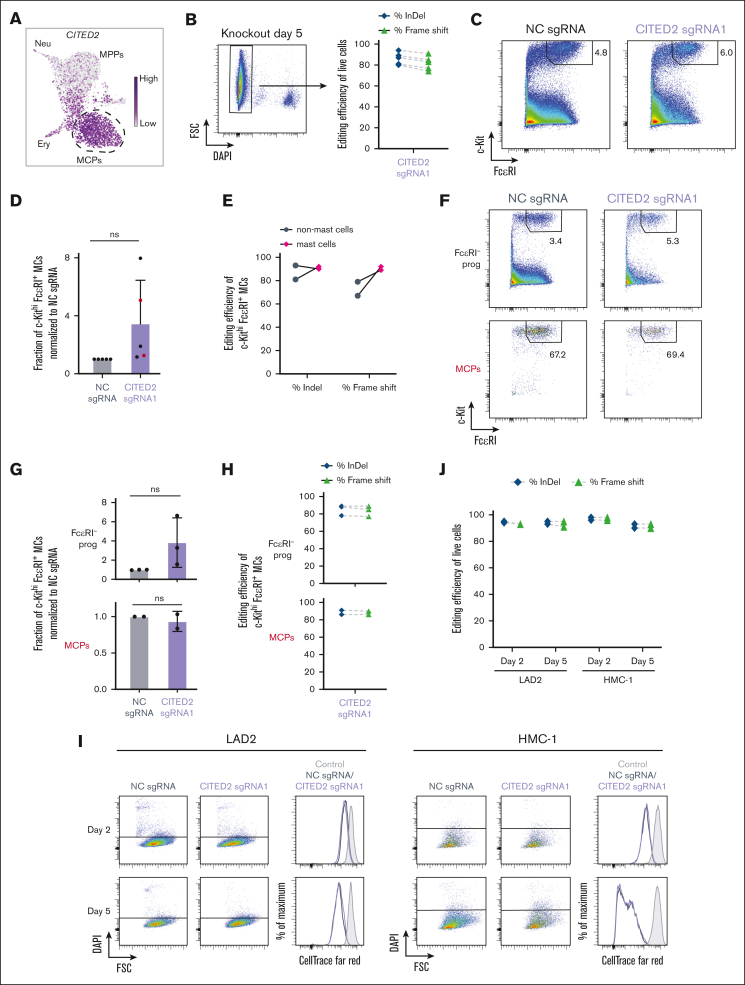
**CITED2 is dispensable for human mast cell differentiation, survival, and proliferation.** (A) Single-cell RNA-sequencing data visualized using the UMAP embedding. The gene expression level of *CITED2* is plotted in the hematopoietic progenitor landscape. (B) Editing efficiency shown as percentage of InDels and percentage of frameshift mutations for CITED2 sgRNA1 of live cells sorted 5 days after electroporation. Five independent experiments were performed. (C) Flow cytometry analysis of mast cells 12 days after electroporation with CITED2 sgRNA1 in CD34^+^ cells. Representative of 5 independent experiments. Live single cells are shown. (D) The fraction of mast cells 11 to 12 days after electroporation. The results show the frequency of c-Kit^hi^ FcεRI^+^ mast cells (among live singlets) in the CITED2 sgRNA1 sample normalized to the corresponding NC sgRNA sample. The cell culture medium was supplemented with IL-3 during the second week in 2 of the 5 experiments. Two-tailed 1 sample *t* test, hypothetical value is 1. The experiments highlighted with red dots were analyzed with regards to editing efficiency in panel E. (E) Editing efficiency of percentage of InDels and percentage of frameshift for CITED2 sgRNA1 of c-Kit^hi^ FcεRI^+^ mast cells and non-mast cells within individual samples sorted 12 days after electroporation. Two independent experiments were performed. (F) Representative flow cytometry plots showing the frequency of mast cells derived from sorted FcεRI^–^ progenitors and MCPs 12 days after electroporation with CITED2 sgRNA1. (G) The fraction of mast cells after electroporation and culture of sorted FcεRI^–^ progenitors and MCPs. The results show the frequency of c-Kit^hi^ FcεRI^+^ mast cells (among live singlets) in the CITED2 sgRNA1 sample normalized to the corresponding NC sgRNA sample. The plots show 3 independent experiments in which CITED2 was knocked out in sorted FcεRI^–^ progenitors and 2 independent experiments in which CITED2 was knocked out in sorted MCPs. Two-tailed 1 sample *t* test, hypothetical value is 1. (H) Editing efficiency shown as percentage of InDel and percentage of frameshift of CITED2 sgRNA1 in c-Kit^hi^ FcεRI^+^ mast cells derived from FcεRI^-^ progenitors (upper panel) and MCPs (lower panel). (I) Flow cytometry plots and histograms showing the survival and the proliferation of LAD2 and HMC-1 electroporated with NC sgRNA and CITED2 sgRNA1, respectively. Representative of 2 independent experiments. Control (gray) refers to all live cells analyzed after staining with CellTrace Far Red and incubating the cells in the fridge. Live single cells are shown. (J) Editing efficiency shown as percentage of InDel and percentage of frameshift in LAD2 and HMC-1 in 2 independent experiments. The NC sgRNA condition refers to nontargeting sgRNA. ns, nonsignificant.

CRISPR-based strategies have successfully been developed to knock out genes in mature in vitro–derived human and mouse mast cells.^11^^,^^12^ Here, we report a vector-free CRISPR/Cas9-based framework using commercially available reagents, electroporation-based delivery, and flow cytometry and Sanger sequencing readouts that allows for determining specific genes’ role in regulating human mast cell differentiation. With the developed approach, we show that *MITF* is required for human mast cell differentiation, whereas *CITED2* is dispensable.

The study was approved by the Swedish Ethical Review Authority (2019-01729).

**Conflict-of-interest disclosure:** The authors declare no competing financial interests.

## Supplementary Material

Supplemental Methods, References, Table, and Figures
