# Supplementary material for: CRISPR/Cas9-mediated gene disruption determines the roles of MITF and CITED2 in human mast cell differentiation
Source: Blood Adv. 2024 Jun 6;8(15):3941–5. doi: 10.1182/bloodadvances.2023012279 (PMC11321385; doi:10.1182/bloodadvances.2023012279)
Supplement: Supplemental Methods, References, Table, and Figures [file BLOODA_ADV-2023-012279-mmc1.pdf]

Supplemental Material

CRISPR/Cas9-mediated gene disruption determines the roles of MITF and CITED2 in human mast cell differentiation

Authors: Jiezhen Mo,[1,*] Fredrik Wermeling,[1] Gunnar Nilsson,[1,2] and Joakim S. Dahlin[1,*,‡]

1. Center for Molecular Medicine, Department of Medicine Solna, Karolinska Institutet and Karolinska University Hospital, Stockholm, Sweden

2. Department of Medical Sciences, Uppsala University, Uppsala, Sweden

* Corresponding authors

Jiezhen Mo, NKS BioClinicum J7:30, Visionsgatan 4, 171 76 Solna, Sweden; e-mail: jiezhen.mo@ki.se

Joakim Dahlin, NKS BioClinicum J7:30, Visionsgatan 4, 171 76 Solna, Sweden; e-mail: joakim.dahlin@ki.se

‡Senior author

Supplemental methods

Ethics statement

The Swedish Ethical Review Authority approved the study (2019-01729).

sgRNA and primer design

The sgRNAs were designed using the websites http://crispor.tefor.net/,[1] http://greenlisted.cmm.ki.se/,[2] and https://chopchop.cbu.uib.no/.[3] The sgRNAs were designed targeting a sequence near the 5' end of the gene. We combined information from the three websites and specifically selected sgRNAs with the highest on-target activity while having a low off-target score. The negative control sgRNA (NC sgRNA) was pre-designed by Sigma-Aldrich. The CD45 sgRNA was designed by Gundry et al.[4] All sgRNAs with stabilizing 2'-O-methyl and phosphorothioate linkages were ordered from Sigma-Aldrich. The sgRNA sequences are shown in supplemental Table 1 and supplemental Figures 2-3.

PCR primers to amplify the sgRNA target region were designed using SnapGene software, and the primers were subsequently ordered from Sigma-Aldrich. The primer sequences are shown in supplemental Table 1.

Cell isolation, genetic editing, and cell culture

Blood mononuclear cells were extracted from buffy coats of anonymous healthy blood donors using Ficoll centrifugation (Cytiva). The CD34 MACS Microbead Kit (Miltenyi Biotec) was used to isolate $CD34^+$ hematopoietic progenitors. Lin^- $CD34^+$ $c\text{-}Kit^+$ $Fc\varepsilon RI^-$ progenitors (hereafter referred to as $Fc\varepsilon RI^-$ progenitors) and Lin^- $CD34^+$ $c\text{-}Kit^+$ $Fc\varepsilon RI^+$ mast cell progenitors (referred to as MCPs) were isolated as described in Wu et al.[5] Briefly, the CD117 MACS Microbead Kit (Miltenyi Biotec) was used to enrich $c\text{-}Kit^+$ cells. $Fc\varepsilon RI^-$ progenitors and MCPs were then sorted on the FACS Aria Fusion system (BD Biosciences). The hematopoietic progenitor cells were cultured in complete StemPro-34 serum-free medium (Thermo Fisher Scientific) containing 2 mM L-glutamine (Cytiva HyClone), 100 U/ml penicillin (Cytiva HyClone), and 0.1 mg/ml streptomycin (Cytiva HyClone). No antibiotics were added to the culture medium immediately after electroporation. Instead, antibiotics were added 2-5 days after the electroporation. The culture medium for the primary cells was supplemented with 5 ng/mL SCF (Swedish Orphan Biovitrum), 50 ng/mL IL-6 (PeproTech), and 10 ng/mL IL-3

(PeproTech) during the first week. The cells were cultured without IL-3 during the second week unless otherwise specified in the figure legend.

LAD2 cells were cultured in complete StemPro-34 serum-free medium containing 2 mM L-glutamine, 100 U/ml penicillin, and 0.1 mg/ml streptomycin, 100 ng/ml SCF added freshly.

HMC-1 cells were cultured in IMDM medium containing 10% FBS, 2 mM L-glutamine, 1.2 mM α-thioglycerol, 100 U/ml penicillin, and 0.1 mg/ml streptomycin.

To form the ribonucleoprotein (RNP), TrueCut™ Cas9 Protein v2 (Thermo Fisher Scientific) and synthetic sgRNA were added to a PCR tube at a molar ratio of 1:3, and the mixture was incubated in a PCR machine at 25°C for 30 minutes. The Amaxa™ Nucleofector™ II System (Lonza) was used to electroporate cells to deliver the Cas9 RNPs. The Amaxa human CD34+ cell nucleofector kit (Lonza) and the program X-001 were used to electroporate the hematopoietic progenitors. The Amaxa cell line nucleofector Kit V (Lonza) and the program T-020 were used to electroporate the cell lines.

Quality control

FACS isolation of a least 10 000 primary hematopoietic progenitors from a given sample was used as a result analysis criterion. One control sample failed to produce a population of mast cells following culture, which made it impossible to study the effect of gene disruption on the formation of mast cells in this sample, and was therefore excluded.

Flow cytometry

Cells were analyzed using the FACS Canto II Cell Analyzer or the FACS Aria Fusion system (BD, Biosciences). FlowJo software (TreeStar, Ashland, OR) was used to analyze the resulting data. Antibodies used for the staining of cells and analyzed by flow cytometry included: CD3 (clone SK7), CD14 (M5E2), CD15 (W6D3), CD19 (HIB19), c-Kit (A3C6E2), FcεRI (AER-37, CRA-1), CD34 (581), and CD45 (HI30). DAPI was used as a viability dye. CellTrace Far Red (Thermo Fisher Scientific) was used to trace proliferating cells.

Sanger sequencing, Inference of CRISPR Edits (ICE) analysis

The cells of interest were sorted into 250 µL or 500 µL of lysis buffer for the extraction of genomic DNA using the QuickExtract™ DNA kit (Lucigen). To amplify the sgRNA target

region, 5 µL genomic DNA template was used in the PCR process. The PCR amplicons were purified using the Wizard SV Gel Clean-up system (Promega) and subsequently submitted to Eurofins Genomics for Sanger sequencing. The obtained Sanger sequencing data was analyzed using ICE (Inference of CRISPR Edits) (Synthego, https://ice.synthego.com)[6] to assess the editing efficiency.

Single-cell RNA-sequencing analysis

Single-cell RNA-sequencing data generated in Wu et al,[5] as deposited in the Gene Expression Omnibus database (GSE184351), was processed and the expression of individual genes was visualized using the UMAP embedding.

Statistical analysis

Statistical analysis was performed using Prism 9 software (Graphpad software, La Jolla, CA). $P < 0.05$ was considered significant. The statistical tests used are specified in the figure legends. The error bars in the figures indicate Standard Deviation (SD).

Reference

1. Concordet J-P, Haeussler M. CRISPOR: intuitive guide selection for CRISPR/Cas9 genome editing experiments and screens. *Nucleic Acids Res*. 2018;46(W1):W242–W245.
2. Panda SK, Boddul S V., Jiménez-Andrade GY, et al. Green listed—a CRISPR screen tool. *Bioinformatics*. 2017;33(7):1099–1100.
3. Labun K, Montague TG, Krause M, et al. CHOPCHOP v3: expanding the CRISPR web toolbox beyond genome editing. *Nucleic Acids Res*. 2019;47(W1):W171–W174.
4. Gundry MC, Brunetti L, Lin A, et al. Highly efficient genome editing of murine and human hematopoietic progenitor cells by CRISPR/Cas9. *Cell Rep*. 2016;17(5):1453–1461.
5. Wu C, Boey D, Bril O, et al. Single-cell transcriptomics reveals the identity and regulators of human mast cell progenitors. *Blood Adv*. 2022;6(15):4439–4449.
6. Conant D, Hsiau T, Rossi N, et al. Inference of CRISPR edits from Sanger trace data. *CRISPR J*. 2022;5(1):123–130.

Supplemental Table 1. Primer and oligo sequences

Oligo name	**Sequence (5' – 3')**	**Description**
MITF forward	GCCTTGATGCAATCAAGCTGAC	PCR primer
MITF reverse	GGAACATTGACTCCCACCATTTACTG	PCR primer
CITED2 forward	GTGGCGCGGTCTCATTATC	PCR primer
CITED2 reverse	CCTGCAGCAGGGTGCAAATC	PCR primer
CD45 sgRNA	GTGCTGGTGTTGGGCGCAC	CD45 sgRNA spacer
MITF sgRNA1	ATACTGGAGGAGCTTATCGG	MITF sgRNA1 spacer
MITF sgRNA2	CGTACCTTAAGGACTTCCAT	MITF sgRNA2 spacer
CITED2 sgRNA1	ATGGGCGAGCACATACACTA	CITED2 sgRNA1 spacer
CITED2 sgRNA2	CGAGCACATACACTACGGCG	CITED2 sgRNA2 spacer
CITED2 sgRNA3	GCGGCATCAGGCATGCGATG	CITED2 sgRNA3 spacer
Negative control sgRNA	CGCGATAGCGCGAATATATT	non-target sgRNA spacer

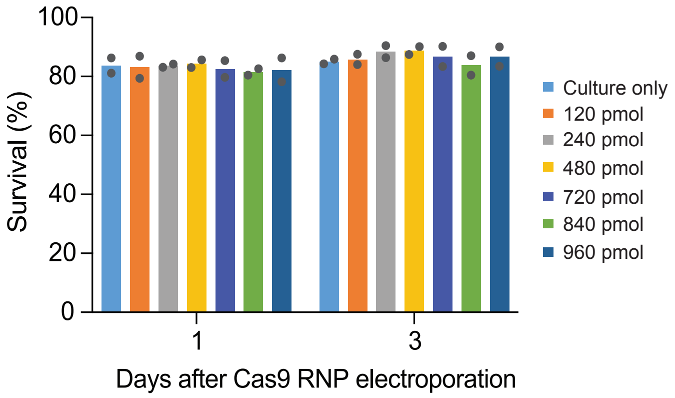

Supplemental Figure 1. The survival was determined by trypan blue staining of $CD34^{+}$ cells electroporated with different dose of Cas9 RNP. Data from two independent experiments are shown in the graphs.

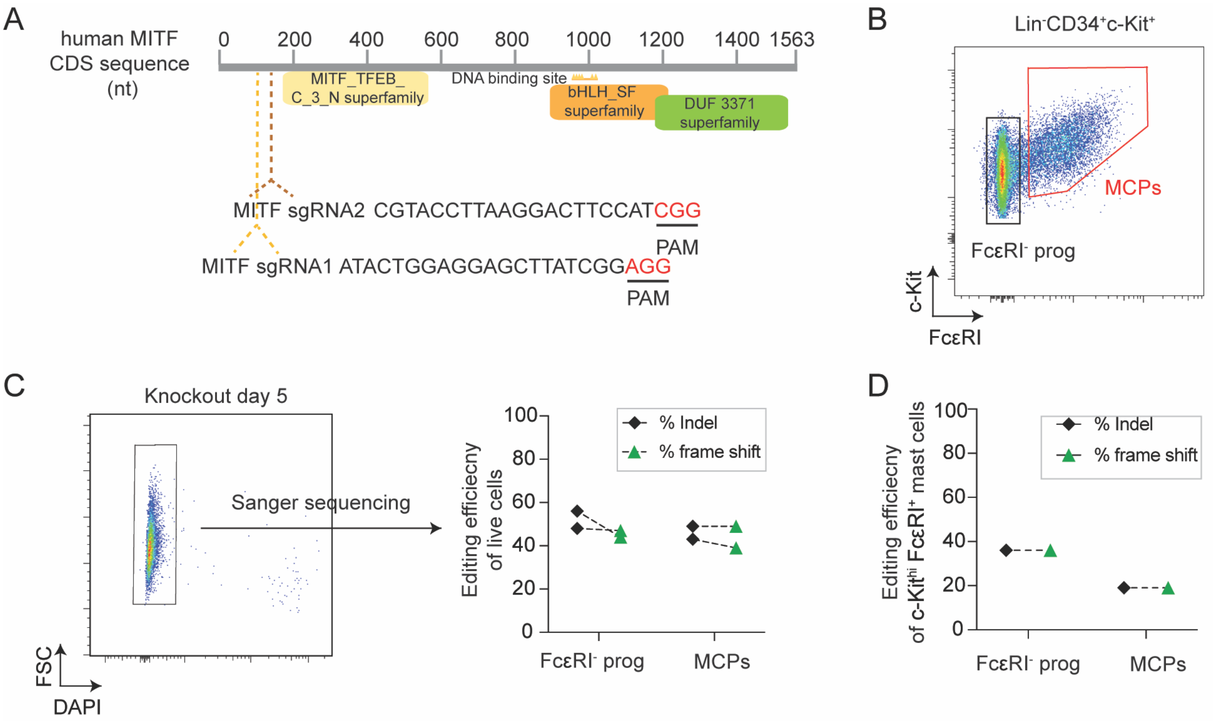

Supplemental Figure 2. (A) Schematic illustration of two sgRNAs target on human MITF. The functional domains of MITF are highlighted. (B) Flow cytometry plots showing the gate of the FcεRI- progenitors (FcεRI- prog) and mast cell progenitors (MCPs). Cells gated on live singlets. (C) Editing efficiency in cultured FcεRI- progenitors and MCPs electroporated with MITF sgRNA2, with the dosage of 840 pmol Cas9 RNP. Live singlets were sorted for Sanger sequencing on KO day 5. (D) Related to Figure 1L, Exp 1. Editing efficiency of mast cells. Mast cells were sorted for Sanger sequencing on KO day 12.

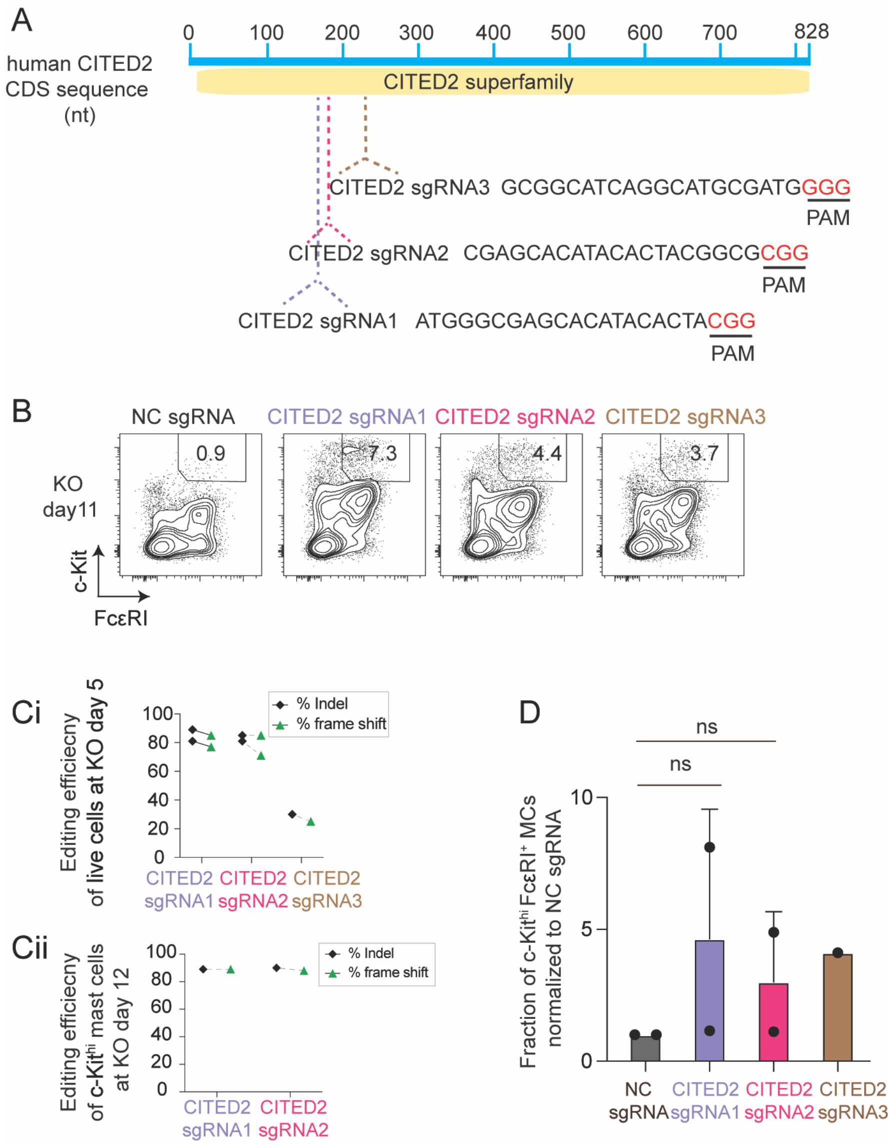

Supplemental Figure 3. (A) Schematic illustration of the CITED2 sgRNA location. (B) Flow cytometry plots showing the gating strategy of cultured CD34$^+$ cells electroporated with negative control (NC) sgRNA and three CITED2 sgRNAs. Cells gated on live singlets. (Ci) Editing efficiency of sorted live singlets 5 days after electroporation. Two independent experiments were performed for CITED2 sgRNA1 and CITED2 gRNA2. One experiment was performed for CITED2 sgRNA3. The sgRNA1 data points are also shown in Figure 2B. (Cii) Editing efficiency of sorted mast cells 12 days after electroporation. (D) Fraction of mast cells normalized to NC sgRNA 11 days after electroporation. The sgRNA1 samples presented here are also shown in Figure 2D. The NC sgRNA condition refers to non-targeting sgRNA. Two-tailed one sample t test, hypothetical value is 1. ns = non-significant.
